# Some Points in the Pathology of General Paralysis of the Insane

**Published:** 1884-12

**Authors:** T. Steele Sheldon

**Affiliations:** Superintendent of the Cheshire County Asylum, Macclesfield; Late Assistant Medical Superintendent, Somerset County Asylum, Wells


### SOME POINTS IN THE PATHOLOGY OF GENERAL PARALYSIS OF THE INSANE.

BY

T. Steele Sheldon, M.B. Lond.,

*Superintendent of the Cheshire County Asylum, Macclesfield;*
*Late Assistant Medical Superintendent, Somerset County Asylum, Wells.*

---

The discovery of the important diagnostic significance of the tendon-reflexes has enabled us to review with profit our knowledge of many diseases of the nervous system, amongst them General Paralysis of the Insane. Owing, probably, to the interesting combination of mental and physical signs, this disease has always possessed special attractions in the eyes of alienists, and many are the knights in pathology who have broken a lance over it; notwithstanding, its pathology is still obscure, and, for peculiar reasons, likely to remain so for some time. One difficulty lies in the fact that opportunity of examination of the nervous centres generally arrives only when the morbid process has done its worst, the quantity of structural mischief then disguising the quality. On the other hand, we are, I believe, almost entirely ignorant of the first expressions of the disease, a distinct feature of which is subtlety of advent. As yet its earliest recognition depends upon signs which point already to serious structural defects initiated, probably, some considerable time previously. I will, indeed, suggest that in cases of ordinary duration *after* diagnosis, the morbid process has existed for at least as long a period *before* diagnosis—the diagnosis resting upon such signs as pupillary changes, slight defects of articulation, moral slidings (often most early observed), &c. In advance of older observers, we are now able to discover tabes dorsalis before the stage of locomotor ataxy—in some cases, many years before—and I believe extended observations will do us similar service in the case of general paralysis, which has many interesting points of contact with tabes dorsalis. Such observations must, however, proceed in the main from the general practitioner of medicine, in whose training lesions of the nervous system have hitherto had sadly too small a place. The ability to detect the disease at an early period could not but throw some light on its pathology, and might give indications for the treatment of an affection which, because it is common and commonly fatal, deserves the closest attention of every student. Here I may be allowed to suggest a line of enquiry: What part does syphilis play in the production of general paralysis? Dowse (*Syphilis of the Brain*, p. 142) makes the following forcible statement: "I should say that syphilis, by the diffuse changes which it is known to instigate in the small vessels of the brain, is the cause of at least two-thirds of the general paralysis which leads to dementia, as we meet with it in this country." This, in my opinion, overstates the case. Of patients drawn from the agricultural class chiefly, I have seen but two, or perhaps three, whose affection could be attributed to syphilis, and it may be questioned whether in these cases the disease ought not to be called syphilis of the frontal lobe, which most writers hold should not be confounded with general paralysis. Without a history of syphilis, however, I believe attempts to distinguish between the two would often fail; and Dowse, commenting on a case of convolutional degeneration of the anterior lobes following syphilis, italicizes the following statement: "This case is one of that series which comes under the title of general paralysis of the insane." (*Loc. cit.*, p. 136.) He proceeds to say that in seven out of twelve such cases under his care during four years, there was a distinct syphilitic history. The point is worthy of notice by those under whose care cases of general paralysis first come. As a rule, patients only find their way to an asylum when the disease is advanced; it is then difficult to obtain any previous history, and treatment avails nothing. I give brief notes of a case under observation at the Somerset Asylum; it is probable, looking at the previous history, symptoms, and *post-mortem* appearances, that the patient was suffering from syphilis.

W. H. B., aged 43, was transferred in September, 1882, from the Gloucester Asylum, where he had been sent as a wandering lunatic. He was in the navy for twenty years; recently he had been out of employment, and had undergone much mental anxiety. His habits were said to be sober. No further history was obtained, except from the patient himself, who stated he had had venereal disease badly; the state of his mind precluded him from entering into details. His condition on admission was as follows:

His expression is dull; during conversation he tends to be emotional; what he says is coherent, but he is unable to sustain conversation, his memory being very poor, especially for dates; he speaks slowly and with difficulty, the facial muscles trembling meantime; the tongue is protruded with a jerk to the right side, and is somewhat tremulous; the pupils are irregular in outline, equal, tending to dilatation; they react to light; his gait is weak; the sphincters are paralysed.

Three months afterwards the symptoms were more serious: utterance is very thick and slow, and he slurs words; as far as it goes, his conversation is coherent. The paresis is very marked: he stoops, sitting and standing; in the latter position, he needs to support himself by his hands on a table, and he walks with great difficulty; he can preserve equilibrium with closed eyes; there is marked tremor of the hands. The paresis is more marked on the left side; on this side the knee-jerk is certainly exaggerated. There have been no convulsions or apoplectiform attacks—simply progressive paresis and dementia. He died a month afterwards.

*Autopsy.*—Body well nourished; no scars, no enlarged glands; calvarium dense, especially in front. On the inner surface of the left os frontis there seems to have been a new deposit of bone, much increasing the thickness of the calvarium in this region; cerebro-spinal fluid excessive. The brain weighs 47½ oz., and is very soft; the pia-arachnoid is very opaque over the anterior half of the convex aspect of both hemispheres, thick, and in some parts of milky whiteness. The right hemisphere is more involved; the pia mater is adherent to a great part of the first transverse frontal gyrus, to the central end of the third frontal and adjoining portion of the ascending frontal; the lips of the sylvian fissure are adherent anteriorly. The gyri generally are much wasted. The basal vessels are slightly thickened, and of bluish opacity. (The smaller vessels were, unfortunately, not examined for the changes described by Heubner.) The cord is firm and, to the unaided eye, healthy.

The heart weighs 10 oz.; the valves are competent; on section, the vessels in the left ventricular wall are seen surrounded by a large white halo, the appearance suggesting a new growth until the lumen of the vessel was found.

The capsule of the liver is thickened at isolated spots, and immediately under the surface at one point is a dark-grey, mottled nodule, not distinctly defined from the surrounding tissue, and of the size of a filbert.

We are now beginning to understand that the spinal cord plays an important *rôle* in general paralysis. Until recently it has been too commonly assumed that the cerebral hemispheres are the only or chief seat of the disease. Boyd and Bucknill long ago expressed their belief that the cord participated, but at that time microscopy spoke faintly, and it is comparatively lately that Westphal's observations have shown the correctness of their assertions. In this direction, most interesting relations are found to exist between general paralysis and other systematic lesions of the nervous system. Briefly, Westphal found: 1st. Affection of the posterior columns throughout their whole length. 2nd. Affection of the posterior portion of the lateral columns. 3rd. Affection of both posterior and lateral columns combined. I have myself found the above lesions in several cases; and I believe that were the cord more frequently examined, they would be found in a large proportion of cases. Hitherto they have received but little attention, notwithstanding their important bearings, clinically and pathologically. Here I would point out the value of a systematic examination of the deep reflexes in general paralysis, and, indeed, most other affections. The knee-tap is a sign requiring neither skill nor time to test, and its presence or absence, particularly the latter, will often throw bright light on a case. Dowse observes that he has often seen cases in the pre-ataxic stage of tabes dorsalis mistaken for nervous dyspepsia, nervous debility, exhaustion, hypochondriasis, &c., when a slight tap on the patellar tendon would have prevented error. Conversely, I have seen a case diagnosed as locomotor ataxy which examination of the knee-jerk, followed up by closer examination of the symptoms, showed to be one of intracranial disease. The deep reflexes in general paralysis are altered more frequently than is generally supposed. One of my late colleagues, Dr. Beatley, has dealt very fully with this question in a graduation thesis, the publication of which I shall not anticipate further than by stating that in a large proportion of the cases he examined the deep reflexes were altered, and that, when so altered, the cases were divided almost equally into those whose patellar reflex was exaggerated and those in which it had disappeared. During the past three months I have admitted three cases of general paralysis into this asylum: in one, the knee-jerk is distinctly exaggerated; in a second, the knee-jerk is excessive, and accompanied by ankle-clonus as marked as I have ever seen it; in the third, the knee-jerk is absent. From former experience, I expect to find double lateral sclerosis in the second, and posterior sclerosis in the third case; in the absence of ankle-clonus, the exaggerated knee-jerk in the first case is not conclusive of lateral sclerosis. Now, sclerosis of the posterior part of the lateral columns is known to occur under the following circumstances: (1) interruption of the pyramidal tract by a focal lesion at any point before its entrance into the cord, the sclerosis being then most usually single; (2) a transverse lesion of the cord by injury or disease within or without the cord, the sclerosis being then probably double; (3) the disease known as *amyotrophic lateral sclerosis;* (4) that little-known disease, *primary lateral sclerosis.* Can the descending sclerosis of general paralysis be brought under any of these heads?

The condition of the brain and cord in one of my cases, in which there were increased knee-jerks and ankle-clonus, was briefly as follows: The pia-arachnoid was opaque and thick over the convex surface of the hemispheres, especially so over the frontal lobes and central gyri; there were no adhesions of pia mater to gyri, but the latter were much wasted; on section, the grey matter was found very thin; *no focal lesion could be found anywhere in the hemispheres, ganglia, pons, or medulla;* to the unaided eye there was distinct sclerosis of both lateral columns of the cord throughout their whole length. Such a case as this does not appear to come under any of the first three divisions, and we seem reduced to the conclusion that the convolutional lesion and the spinal sclerosis are independent events, explaining the frequent coincidence by saying that in general paralysis there is a tendency to the deposit of fibrous tissue throughout the nervous centres. A fatal objection to such explanation lies in the question, "Why should the lateral columns only display this tendency?" I suggest with diffidence another explanation: It is now believed that the pyramidal fibres are basal prolongations of certain giant cells in the third layer of the cortex in the Rolandic region of the hemispheres. Separation of the fibres from the influence of the cells, as happens when a hæmorrhage occurs in the internal capsule, leads to the change known as secondary degeneration. In general paralysis these cells suffer in common with other components of the cortex: "The larger cells further in, and the large multipolar cells, are more or less degenerated or atrophied, especially in patches and areas." (Clouston *on Mental Diseases*, p. 377.) Now, looking at the effect upon the issuing fibres of destruction or atrophy of the large cells of the anterior cornua of the cord, it appears to me that a similar effect might be expected in the case of destruction of the cells originating the fibres of the pyramidal tract, and that the sclerosis in cases of general paralysis where no focal lesions can be found might arise in this manner. "It is not yet proved that destructive lesions limited to the grey cortical matter of the Rolandic region are productive of secondary degenerations. Nevertheless, some facts taken from the pathological anatomy of general paralysis tend to show that this is really the case." (Charcot *on Localisation of Cerebral and Spinal Disease*, New Syd. Soc., p. 196.) *

More difficult of explanation still is the connection between general paralysis and sclerosis of the posterior columns of the cord. Medical literature contains several reports of cases of tabes dorsalis terminating in general paralysis. Clouston (*loc. cit.*, p. 364) relates one and alludes to others; from the tabes point of view, however, the sequence seems to be regarded as rare. Looking from the other side, I believe it is a not infrequent event. Since my attention was directed to the point, I have, during the past two years, met with several cases of general paralysis in which the knee-jerk was absent; and in such as he had the opportunity of examining after death Dr. Beatley found well-marked posterior sclerosis. Invariably, loss of the knee-jerk was the most noteworthy

* Since writing the above, I find that Flechsig interprets the sclerosis as consecutive to cortical lesions. Recently, Westphal has combated this view, holding that it has not yet been demonstrated, and is of opinion that the sclerosis is primary ; indeed, he believes that in general paralysis only has primary lateral sclerosis yet been described, for in Morgan and Dreschfeld's celebrated case the anatomical appearance pointed rather to amyotrophic lateral sclerosis than to primary lateral sclerosis. Lateral sclerosis occurs too frequently in general paralysis to allow one to regard it merely as a coincidence, and herein, I think, lies a serious objection to Westphal's view, which affords no explanation of the association between the lesions of the brain and the lateral columns of the cord.

sign of such lesion, other symptoms being apparently masked by the paralytic and mental state. There was nothing in the previous history (scanty as it generally was) of the cases pointing to early tabic symptoms. The only instructive history I have got was given by a patient now under my care. He is a painter and decorator by trade, and has a blue line at the edge of the gums. He denies ever having had colic, having been in the habit of taking a purge weekly. He has hard arteries, and marked arcus senilis. He denies syphilis, but says his wife miscarried four times. Seven years ago he began to suffer from symptoms which continued during three years: one was giddiness, which proved very inconvenient when he had to work on a scaffold. At one time he had dimness of vision, which suddenly disappeared; at intervals during this time he suffered much from *peculiar pains about the waist and in the legs, which he described by darting his finger rapidly forwards.* He is now unmistakeably suffering from general paralysis. I believe that in such cases as these the cord lesion is primary, though often undetected. As mentioned above, the pre-ataxic symptoms of tabes dorsalis are probably often misinterpreted, and to the disadvantage of the patient; for, if attacked early, the disease does not appear to be so obstinate as has been supposed. (Dowse, *loc. cit.;* Althaus, *Sclerosis of the Spinal Cord.*) In this connection, the importance of syphilis as a cause of tabes dorsalis will be at once observed. There is still a "missing link" between posterior sclerosis and convolutional degeneration. So far as I know, there is no detailed account published of the lesion of the cord being traced into the hemispheres. Clouston (*loc. cit.*, p. 364), indeed, says he has traced the spinal disease up through the medulla and the lower ganglia into the brain cortex; but an *ex cathedrâ* statement cannot be fully accepted when we come to consider the complicated anatomical relations which exist between the posterior columns of the cord and the cortex—relations still ill-understood. It is as difficult to explain the association as to say why atrophy of the optic nerves, or paralysis of the oculo-motor nerves, should accompany tabes dorsalis.

An important pathological conclusion from the foregoing is, that two cases of general paralysis may differ *toto cœlo* in their genesis; whilst in one case the morbid process may *commence* in the cortex, in the other it may *terminate* there. It does not lie within the scope of this paper to discuss the difference between the symptoms of these two varieties, and I shall not do more than mention that in many cases there is no well-defined cord-lesion found, agreeing with the absence of change in the deep reflexes during life.

I do not suppose that I have written anything which is not more or less familiar to those who have a large experience of general paralysis and a knowledge of its literature. My object has rather been such that this paper might have been entitled, "A Plea for a closer Investigation of the Causes and early Symptoms of General Paralysis by my brethren in general practice."

